# Interpretable correlation descriptors for quantitative structure-activity relationships

**DOI:** 10.1186/1758-2946-1-22

**Published:** 2009-12-24

**Authors:** Benson M Spowage, Craig L Bruce, Jonathan D Hirst

**Affiliations:** 1 School of Chemistry, University of Nottingham, University Park, Nottingham, NG7 2RD UK; 2 AstraZeneca, Mereside, Alderley Park, Macclesfield, Cheshire SK10 4TG UK

**Keywords:** Angiotensin Converting Enzyme, Partial Little Square, Angiotensin Converting Enzyme Inhibitor, QSAR Modelling, Enalaprilat

## Abstract

**Background:**

The topological maximum cross correlation (TMACC) descriptors are alignment-independent 2D descriptors for the derivation of QSARs. TMACC descriptors are generated using atomic properties determined by molecular topology. Previous validation (*J Chem Inf Model* 2007, **47**: 626-634) of the TMACC descriptor suggests it is competitive with the current state of the art.

**Results:**

Here, we illustrate the interpretability of the TMACC descriptors, through the analysis of the QSARs of inhibitors of angiotensin converting enzyme (ACE) and dihydrofolate reductase (DHFR). In the case of the ACE inhibitors, the TMACC interpretation shows features specific to C-domain inhibition, which have not been explicitly identified in previous QSAR studies.

**Conclusions:**

The TMACC interpretation can provide new insight into the structure-activity relationships studied. Freely available, open source software for generating the TMACC descriptors can be downloaded from http://comp.chem.nottingham.ac.uk.

**Electronic supplementary material:**

The online version of this article (doi:10.1186/1758-2946-1-22) contains supplementary material, which is available to authorized users.

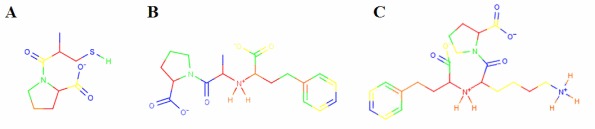

## Background

Quantitative structure-activity relationship (QSAR) models correlate molecular chemical structure to biological activity. The underlying principle for QSAR modelling is the similar property principle: molecules with similar chemical structures will exhibit similar biological properties [1]. This principle can be explained by changes in chemical structure altering the electron distribution within a molecule, which is directly responsible for the activity of the molecule. QSARs can be used to elucidate a quantitative description of changes in biological activity arising from the exchange of the functional groups within a molecule. In general, QSAR modelling requires three main features: a data set of molecules, appropriate descriptors and an efficient statistical method for capturing correlation. Descriptors are characteristic properties of molecules, often represented as numerical values, which facilitate the analysis of chemical structure. A wide variety of molecular descriptors are available and descriptor selection is an integral process in QSAR modelling [2].

2D QSAR models are generated using descriptors derived from the two-dimensional graph representation of a molecule. In contrast, 3D QSAR models correlate activity with descriptors based on spatially localised features. Although 3D descriptors may allow more detailed descriptions of the molecular binding interactions between ligands and receptors, 3D methods are more time-consuming, due to the requirement of precise conformational detail on the molecule and exact alignment [3]. In some cases, 2D QSAR methods can classify the biological activity molecules more efficiently than some more complex 3D QSAR methods [4]. In many instances, the biologically active conformation of a molecule is unknown and 2D descriptors are useful, as they are not dependent upon spatial conformation.

Classic QSAR methods, developed by Hansch [5], provided a foundation on which numerous QSAR methods are now based: the correlation of physicochemical properties to activity using multivariable regression. Regression analysis models the activities of molecules through an equation constructed using a linear combination of physicochemical properties. The coefficient for each variable in the equation can, consequently, be examined to determine the extent to which each property contributes towards the activity of the molecule. Regression is central to many contemporary QSAR methods, although nowadays often the technique of partial least squares (PLS) [6] is used to cope with large numbers of descriptors. One of the appeals of regression is the relative ease with which models can be interpreted and this extends to approaches based on PLS [7]. Sometimes an interpretable model might be favoured over a more accurate, but less transparent, QSAR [8].

Over the last decade, advances in computational technology combined with contemporary methodologies have led to a vast array of new descriptors [2]. Topological maximum cross correlation (TMACC) descriptors were created [9] with the intention of developing an interpretable 2D descriptor for QSAR modelling. The TMACC descriptors are based on concepts derived from the grid-independent descriptors (GRIND) [10]. GRIND are alignment-independent 3D molecular descriptors which represent a molecule using a grid on which the product of pairs of force field interactions is plotted against the distances between the pairs [10]. This method is analogous to the autocorrelation descriptor, which represents atom pairs as a weighted histogram [11]. GRIND are interpretable, as only one value is stored for each distance range: the maximum product of the two force field interactions. This method was termed maximum auto- and cross-correlation (MACC) [10]. In a similar method, the TMACC descriptors use the topological bond distances and physicochemical properties of a molecule. Only the maximum value calculated as the product of pair combinations of physicochemical properties for each distance is used to generate the TMACC descriptors.

Previous validation of the TMACC descriptors was promising, with leave-one-out (LOO) cross-validated correlation coefficients comparable to those achieved by the state-of-the-art 2D QSAR method, hologram QSAR [9]. An external test set is often used to estimate predictive accuracy [12]. However, the external test set must be large to give results as reliable as cross validation [13–15]. We have previously shown [9], on the datasets in this study, that using a training/test set partition gives estimates of predictive accuracy that are qualitatively similar to those from cross validation. Thus, here we use cross validation only, as it makes more use of the data for model building. Whilst statistical validation is key, the interpretation and chemical significance of the structure-activity relationships generated are also important [2, 12]. To assess the interpretive ability of a QSAR model it is necessary to apply scientific rationale to the resultant interpretation [16]. Interpretation of the TMACC descriptors is achieved through analysis of the regression model generated by PLS. The predicted activity of a molecule can be attributed to specific atoms that contribute towards the TMACC descriptors. Visualization of the resultant atom activity contributions is accomplished by atomic colour coding based on sign and magnitude of partial activity.

The present study aims to evaluate the ability to identify known structure-activity relationships using the TMACC descriptors. To exemplify the TMACC descriptors, we investigate two datasets, which were previously used in a comprehensive comparison of modern QSAR approaches [17]. Models derived from the angiotensin converting enzyme (ACE) and dihydrofolate reductase (DHFR) inhibitor data sets were assessed to elucidate the encoded structure-activity relationships with the help of information in the literature and to evaluate the interpretive ability of TMACC models.

### Experimental

The data sets used in this study, 114 inhibitors of angiotensin converting enzyme [18] and 397 inhibitors of DHFR [17], have been widely used to investigate many QSAR methods [17]. Each data set contained experimentally determined activity (pIC_50_) values for each molecule. TMACC descriptors were generated using the topological data of each molecule. All nonpolar hydrogen atoms were removed and their atomic value added to the heavy atom to which they were bonded. Polar hydrogen atoms were considered explicitly. Physicochemical properties were then assigned to each atom. Four parameters were used to represent these properties: Gasteiger partial charges [19], log*S* parameters [20], Crippen-Wildman molar refractivity parameters [21] and Crippen-Wildman partition coefficient (log*P*) parameters [21]. Gasteiger partial charges were calculated using the method of partial equalization of orbital electronegativity [19]. This procedure calculates atomic charges in σ-bonded and non-conjugated π-systems using only the topology of a molecule. Log*S* parameters were used to describe atomic contributions to aqueous solubility [20]. Crippen-Wildman molar refractivity (MR) was used as a measure of the steric effect, which is determined through classification of atoms based on neighbouring atoms [21]. Crippen-Wildman partition coefficients (log*P*) are assigned to each atom as a measure of atomic lipophilicity, determined in the same way as Crippen-Wildman molar refractivity.

Property types which produce positive and negative values were considered as two separate properties (Table 1). This was the case for all property types, except molar refractivity, as all atomic values for this property are positive. To account for the different scales used by each atomic parameter, each contribution was rescaled by the largest absolute value, resulting in all values being confined within the range of +1 and -1.

**Table 1:** Properties used in generation of TMACC descriptors

Physicochemical property	Separate property
Gasteiger partial charge (Electrostatics)	Positive charge
	Negative charge
log*S* (Solubility and solvation phenomena)	Positive log*S*
	Negative log*S*
Crippen-Wildman log*P* (Hydrophobicity)	Positive log*P*
	Negative log*P*
Crippen-Wildman Molar Refractivity (Sterics and polarizability)	Molar refractivity

The TMACC descriptor was derived from the autocorrelation descriptor [11]. The standard equation for calculating an autocorrelation descriptor, *X*_*ac*_, (Equation 1), considers a property, *p*, and the topological distance, *d*, between atoms *i* and *j*:1

TMACC descriptors are calculated as the product of the physicochemical properties as determined for every atom pair within a given molecule. Only the maximum value determined for any bond distance is used in the generation of the TMACC descriptors. All other values are neglected. As the TMACC descriptor incorporates both autocorrelation and cross-correlation, all possible combinations of physicochemical properties are considered. The equation for calculating a TMACC descriptor (*X*_*TMACC*_) (Equation 2) summarises this approach, involving two properties, *p* and *q*, for two atoms, *i* and *j*, separated by the topological distance, *d*:2

Interpretation of the TMACC descriptors was accomplished by rescaling the coefficients from the non-cross-validated model. For every unscaled descriptor, *x*_*i*_, we define the partial activity as *β*_*i*_*x*_*i*_, using the unscaled regression coefficient, *β*_*i*_. This provided a method for identification of the atoms which contribute towards each descriptor for a particular molecule. Each atom contributing to a descriptor was given an equal share of the partial activity. The atom contribution values were subsequently summed for each atom. For a given dataset, the total atom contributions were separated into five activity bands, ranging from 'very negative' to 'very positive' (Table 2), each containing an equal number of atoms. By colour-coding each atom according to its activity band, it was possible to visualize the activity of each atom.

**Table 2:** The activity bands used for TMACC descriptor interpretation

Activity	Threshold		Colour
	ACE	DHFR	
Very Positive	0.27	0.61	Blue
Positive	0.074	0.072	Yellow
Neutral	0.030	0.024	Green
Negative	0.0078	-0.00012	Orange
Very Negative	-0.0033	-0.0089	Red

All calculations were performed using Nottingham Cheminformatics Workbench (NCW), a package which provides the function of generating the TMACC descriptors and the TMACC interpretation. NCW is a Java-based application, which is suitable for all major operating systems. It builds upon the original TMACC code (available from our website http://comp.chem.nottingham.ac.uk/download/tmacc). The original software only generates TMACC descriptors; there is no facility to interpret your model. NCW allows the user to start with a set of molecules and perform a full analysis upon them. The popular machine-learning workbench Weka is included to provide PLS modeling, as well as an in-house implementation. The in-house PLS algorithm was written before Weka included one. The results of the PLS analysis are used to determine the atomic contribution of each atom. The interpretation is visualized by a colour scheme depicting activity contribution by atom. The user is able to view molecules individually or tabulated. NCW will be available as open source software for download from our website, http://comp.chem.nottingham.ac.uk/download/ncw. NCW utilizes Marvin for drawing and manipulating chemical structures: Marvin 5.2.2, 2009 http://www.chemaxon.com. All molecular graphics were created using YASARA ("Yet Another Scientific Artificial Reality Application," http://www.yasara.com).

## Results and Discussion

### Angiotensin converting enzyme (ACE) inhibition

LOO cross-validation of the PLS models generated for the ACE and DHFR data sets gave *q*^2^ values of 0.70 and 0.53, respectively, consistent with those previously reported [9]. TMACC descriptors were generated for the ACE data set of 114 inhibitor molecules, previously used to assess the ability of a 3D QSAR method, comparative molecular field analysis (CoMFA) [18]. The data set contained a diverse range of structures and activities selected from literature [18]. ACE is a zinc metallopeptidase, which functions as a dipeptidyl carboxypeptidase, hydrolysing a range of oligopeptide substrates [22]. It acts to induce hypertension and is, consequently, a widely investigated target for antihypertensive drugs [23]. Several methods used to determine the ACE inhibitory activities (IC_50_ values) involved the use of the substrate hippuryl-histidyl-leucine (HHL) [24, 25]. An early method for determining ACE inhibitor activity was to measure the rate of hippuric acid production from HHL catalyzed by ACE [26]. However, it was subsequently discovered that HHL is a C-terminal domain specific substrate of ACE [27, 28]. Consequently, the structure-activity relationship shown by this data set is likely to reflect that of C-terminal domain specific ACE inhibition, rather than general ACE inhibition.

**Figure 1:**
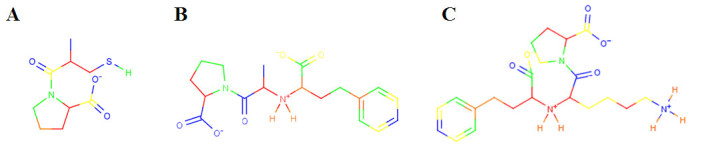
**TMACC interpretation of ACE inhibitors**. TMACC activity colour scheme: red for very negative activity; orange for negative activity; green for neutral activity; yellow for positive activity and blue for very positive activity. A) molecule 87, captopril; the sulfhydryl zinc binding group, P1' methyl group, central carbonyl and the C-terminal carboxylate are all shown in blue, indicating that they provide a positive contribution to the activity of the molecule. B) molecule 64, enalaprilat; the carboxylate zinc binding group, P1' methyl group, central carbonyl and the C-terminal carboxylate are all shown to provide a positive contribution to the activity of the molecule. C) molecule 65, lisinopril; the carboxylate zinc binding group, P1' lysyl group, central carbonyl and the C-terminal carboxylate all provide a positive contribution to the activity of the molecule.

**Figure 2:**
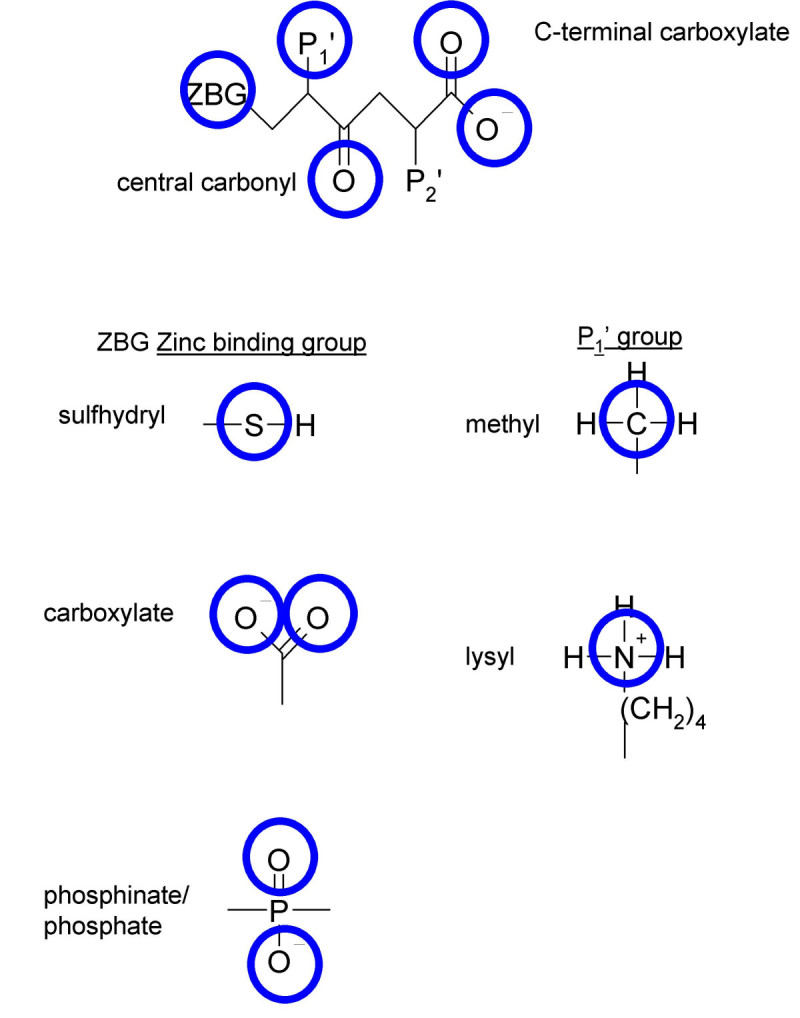
**ACE inhibitor features investigated**. Position of features shown in 2D relation to one another. Blue circles surround atoms studied for activity.

For each molecule, a TMACC interpretation was generated, as described in the Methods section, leading to the labelling of each atom according to an activity banding (i.e., its contribution to activity). Some examples are shown in Figure 1. Based on the literature, several potentially important features (in the form of functional groups) were identified (Figure 2). Using the TMACC interpretation the extent of activity associated with each feature was accumulated for the entire ACE data set to allow determination of the structure-activity relationship modelled (Table 3).

**Table 3:** Frequency of activity of ACE inhibitor features as determined by the TMACC interpretation.

	Activity		
ACE inhibitor feature	Negative	Neutral	Positive
C-terminal carboxylate carbonyl	5	0	105
C-terminal carboxylate hydroxyl	0	5	105
Central carbonyl	13	3	96
Zinc binding carboxylate - carbonyl	3	3	22
Zinc binding carboxylate - hydroxyl	0	4	24
Zinc binding sulfhydryl sulfur	0	0	33
Zinc binding phosphinate phosphorus	0	0	22
Zinc binding phosphinate carbonyl	20	0	2
Zinc binding phosphinate hydroxyl	20	0	2
P1' methyl	4	2	27
P1' lysyl nitrogen	0	0	20

An essential feature of any ACE inhibitor is a zinc coordinating group. The catalytic zinc ion is coordinated by three highly conserved residues present in both somatic ACE (sACE) domains [29]. The important functional role of the zinc ion in the active site domains of ACE has led to the development of peptide based inhibitors, such as enalaprilat, with additional zinc-binding functional groups, including thiol, carboxylate and phosphinate groups. The importance of zinc binding functional groups in ACE inhibition has been demonstrated in crystal structure and structure-activity studies [22, 30, 31].

Zinc binding groups were frequently recognized by the TMACC interpretation as positive for activity. All sulfhydryl sulfur atoms located in the optimal position for zinc-binding were identified as positive for activity. Analysis of phosphinate zinc binding groups showed all phosphorus atoms were identified as positive for activity. However, phosphinyl oxygen atoms were mostly shown as negative for activity. In contrast, the interpretation most frequently identified both carboxylate zinc binding group oxygen atoms to be positive for activity. Although the results do not fully capture the correlation between the type of zinc-ligand and inhibitor activity observed in structure-activity studies, (phosphinate > carboxylate > sulfhydryl) [18], perhaps the negative activity attributed to the phosphinate oxygen atoms reflects its weak zinc-binding ability in comparison to the other zinc binding groups.

**Figure 3:**
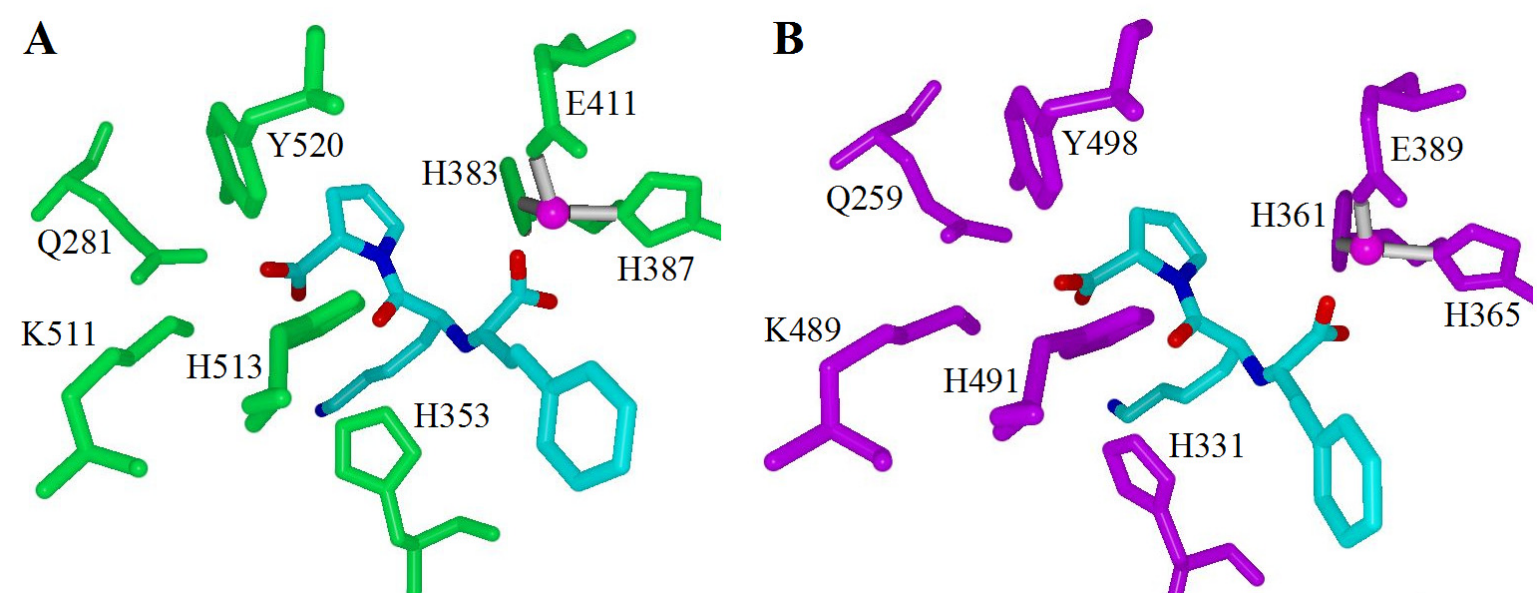
**Conserved ACE residues that interact with lisinopril**. A) tACE active site (green) [22]. B) The N-domain active site of sACE (purple) [29]. Zinc ion shown in magenta; atoms are coloured as follows: red for oxygen, blue for nitrogen, cyan for carbon and grey for hydrogen.

The central carbonyl group is a feature found in most ACE inhibitors. It forms two hydrogen bonds within both domains of ACE [22, 29]. Docking studies suggest this interaction is frequently present in ACE-inhibitor binding [32] and it has been identified in many ACE-inhibitor crystal structure complexes (Figure 3) [33]. Mutation of ^513^His to alanine causes a 120,000-fold decrease in the binding of lisinopril to the C-domain of sACE [34]. This suggests the interaction of the conserved histidine residues with the carbonyl group of an inhibitor is important for ACE inhibition. The TMACC interpretation identified the central carbonyl as favourably contributing towards the activity (Table 3). The high frequency of positive activity shown for this feature by the TMACC interpretation is consistent with the aforementioned literature.

The crystal structures of testicular ACE (tACE) in complex with various inhibitors (Figure 3) show the intermolecular interactions responsible for ACE inhibition in tACE and correspondingly the C-terminal domain of sACE [22, 30, 33]. In contrast to most zinc protease inhibitors, which primarily rely on the strength of their zinc binding groups for activity, domain-specific ACE inhibitors utilize weak zinc binding groups and exploit both primed and unprimed sides of the active site in order to mimic peptide substrates, thereby achieving domain selective inhibition [35]. Domain-specific inhibition of ACE is important, as each domain possesses individual functions [36]. This discovery has developed the number of applications of ACE inhibitors, extending from treating hypertension to protecting stem cells during chemotherapy [37]. A recent study has also suggested ACE may be involved in many physiological processes other than blood pressure regulation [38].

The two domains of sACE contain many conserved residues, which are vital for substrate and inhibitor binding (Table 4). The identification of conserved residues within ACE and their role in inhibitor binding has highlighted several important features required for ACE inhibition, providing a rationale for the structure-activity relationship of ACE inhibitors.

**Table 4:** Conserved ACE residues important for inhibitor interactions

Functional interaction	C-domain residue	N-domain residue
Zinc-binding	^383^His	^361^His
Zinc-binding	^387^His	^365^His
Zinc-binding	^411^Glu	^389^Glu
Inhibitor carbonyl hydrogen bonding	^513^His	^491^His
Inhibitor carbonyl hydrogen bonding	^353^His	^331^His
Inhibitor carboxy terminal carboxylic ionic bonding	^511^Lys	^489^Lys
Inhibitor carboxy terminal carboxylic hydrogen bonding	^281^Gln	^259^Gln
Inhibitor carboxy terminal carboxylic hydrogen bonding	^520^Tyr	^498^Tyr

(Table formulated using information from [29, 30, 32])

A C-terminal carboxylate is found in many ACE inhibitors. This feature interacts with several conserved residues in both domains of sACE, hydrogen bonding with tyrosine and glutamine residues, and also forms an electrostatic interaction with a lysine residue (Figure 3) [32]. Both C-terminal carboxylate oxygen atoms were identified as positive by the TMACC interpretation (Table 3).

**Figure 4:**
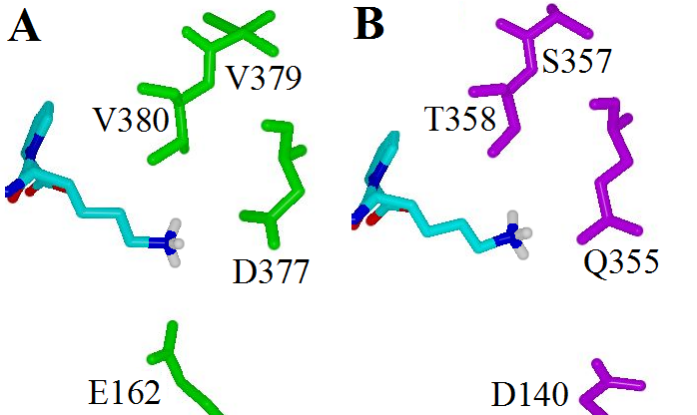
**Comparison of the S1' sub-site residues which bind the lysyl group of lisinopril**. A) tACE (green) [22] and B) the N-terminal domain of sACE (purple) [29]. Colour scheme is identical to Figure 1.

Despite the high level of conserved residues present in both domains of sACE, variation between the domains confers different substrate and inhibitor preferences. The presence of hydrophobic residues ^379^Val and ^380^Val in the S1' sub-site of the C-domain of sACE provides hydrophobic interactions between the sub-site and the P1' residue of inhibitor molecules, such as the P1' methyl group of captopril and enalaprilat [29]. The corresponding residues found in the N-terminal domain, ^357^Ser and ^358^Thr, provide a polar environment and, therefore, do not form similar hydrophobic interactions with the P1' residue of inhibitors [29, 33]. In the C-terminal domain the lysyl chain of lisinopril extends into the S1' sub-site and forms an electrostatic interaction with ^162^Glu and a water-mediated interaction with ^377^Asp [22]. However, in the N-terminal domain the S1' sub-site makes fewer contacts with the lysyl chain of lisinopril (Figure 4). For example, ^162^Glu (C-domain) is replaced by ^140^Asp (N-domain), and due to the larger distance between the lysyl chain and this residue, no electrostatic interaction is observed at this location in the N-domain [29]. Additionally, ^377^Asp (C-domain) is replaced by ^355^Gln (N-domain), thereby abolishing the water-mediated interaction shown between the lysyl residue of lisinopril and the C-domain [29]. This evidence suggests that methyl and lysyl groups located in the P1' position of ACE inhibitors can form favourable interactions with the S1' sub-site of the C-terminal domain of sACE.

Interestingly, the TMACC interpretation identified all inhibitor P1' lysyl nitrogen atoms as favourably contributing towards activity (Table 3). The interpretation also identified inhibitor P1' methyl groups as positive for activity. Thus, the TMACC interpretation identified P1' groups important for C-domain specific ACE inhibition, as illustrated in Figure 1. This C-domain specific bias in the data set, reflected by the TMACC interpretation, has not been shown in previous QSAR investigations using this data set [17, 18].

### Dihydrofoloate reductase (DHFR) inhibition

**Figure 5:**
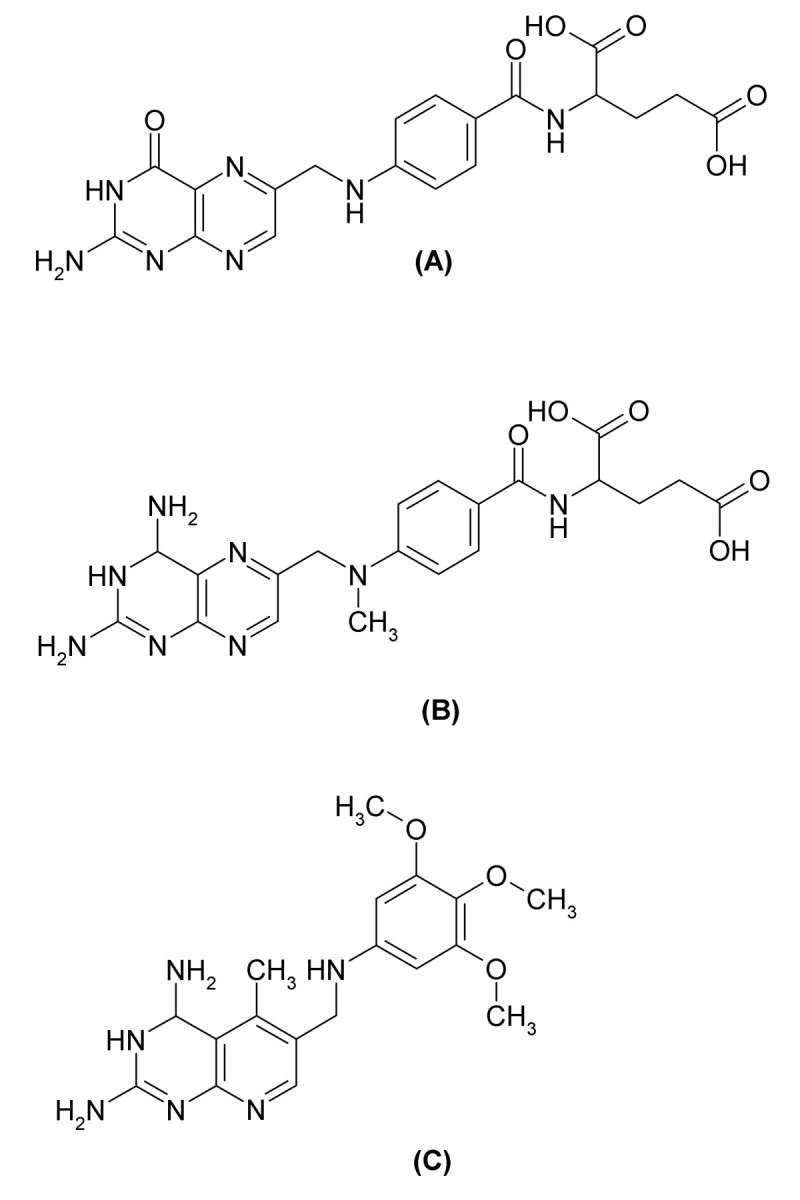
**Chemical structures of folates and antifolates**. A) folic acid (DHFR substrate). B) methotrexate (classical antifolate DHFR inhibitor). C) trimetrexate (non-classical antifolate DHFR inhibitor).

Due to the vital cellular function and ubiquitous expression of DHFR, inhibitors of this enzyme have been used clinically in the treatment of a range of diseases [39]. A variety of antifolates, which inhibit specific DHFR enzymes, have clinical application against cancer, malaria and many infectious diseases caused by bacteria, fungi and protozoa [40]. Natural folates contain a pteridine ring system, a p-aminobenzoic acid and a glutamate moiety (Figure 5). Classical antifolates are analogous to natural folates, possessing a glutamate residue, which allows polyglutamylation catalyzed by folylpolyglutamate synthetase [41]. Methotrexate (MTX) is a classical antifolate, which has been used clinically as an anticancer drug for over 50 years [42]. MTX is a potent inhibitor of DHFR from many species. However, it is restricted to anti-tumour applications, as a consequence of the dependence of classical antifolates on folate carrier-mediated transport, which is found only in mammalian cells [43]. Trimetrexate (TMQ) is a potent non-classical antifolate DHFR inhibitor, which is used in the treatment of *Pneumocystis* infections common in AIDS patients [44].

**Figure 6:**
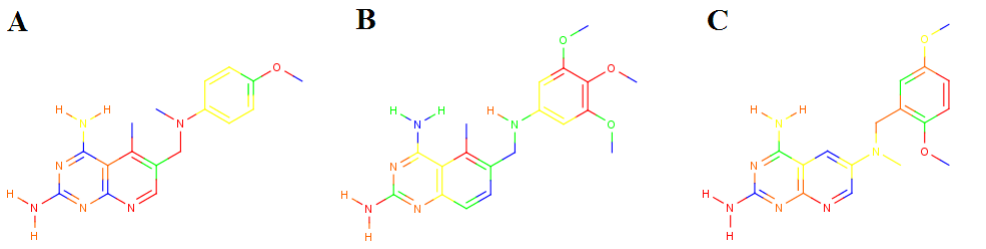
**TMACC interpretation of DHFR inhibitors**. TMACC activity colour scheme: red for very negative activity; orange for negative activity; green for neutral ctivity; yellow for positive activity and blue for very positive activity. A) Molecule 202; the 2-amino group, N1 nitrogen and N8 nitrogen are shown to provide a negative contribution to the activity of the molecule. The 4-amino group, 5 methyl, 10 methyl and the methyl group of the benzylmethoxy are all shown to provide a positive contribution to the activity of the molecule. B) molecule 22, trimetrexate; the 2-amino group and N1 nitrogen are both shown to provide a negative contribution to the activity of the molecule. The 4-amino group, 5 methyl, C8 carbon and the methyl groups of the benzylmethoxy groups are all shown to provide a positive contribution to the activity of the molecule. C) molecule 189; the 2-amino group, N1 nitrogen and N8 nitrogen are shown to contribute negatively to the activity of the molecule. The 4-amino, 9 methyl and the methyl groups of the benzylmethoxy groups are all shown to provide a positive contribution to the activity of the molecule.

**Figure 7:**
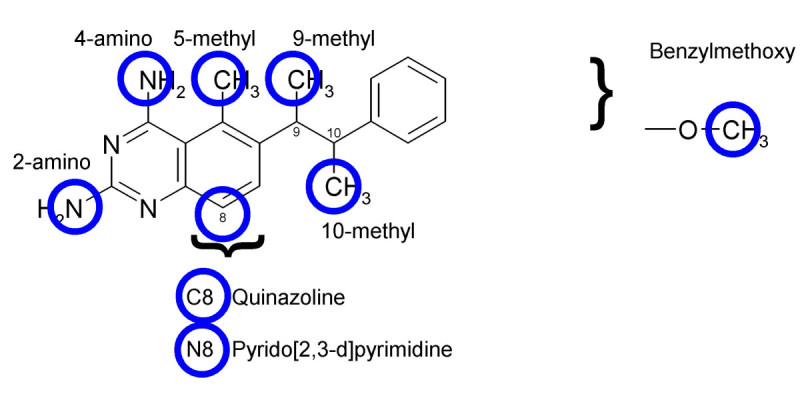
**DHFR inhibitor features investigated**. Position of features shown in 2D relation to one another. Blue circles surround atoms which were studied for activity. Methoxy groups located at all positions of the benzyl ring were studied.

TMACC descriptors were generated for the DHFR data set of 397 molecules, previously studied [17]. The inhibitor activity values for the DHFR data set represent inhibitor potency against rat liver DHFR. Rat liver DHFR shows a high level of conservation of both primary sequence and active site residues and, therefore, is used as a mammalian standard for DHFR inhibition [45]. Following the strategy adopted for the ACE inhibitors, TMACC interpretations were developed for the DHFR inhibitors (Figure 6). Based on the literature, several DHFR inhibitor features were identified (Figure 7) and the association of each feature with activity was extracted from the TMACC interpretation and accumulated for the entire data set (Table 5).

**Table 5:** Analysis of the TMACC interpretation of the DHFR data set.

	Activity		
DHFR inhibitor feature	Negative	Neutral	Positive
2-amino nitrogen	155	81	161
4-amino nitrogen	110	61	212
N1-nitrogen	307	58	32
C8 Quinazoline	0	5	45
N8 Pyrido [2,3-d]pyrimidine	68	16	11
5-methyl	1	0	64
9-methyl	0	1	16
10-methyl	10	3	57
Benzylmethoxy - methyl	5	6	392

Table shows frequency of activity of DHFR inhibitor features as determined by the TMACC interpretation

Crystal structures of DHFR-inhibitor complexes for many species have revealed conserved binding residues and have provided a basis for the design of selective DHFR inhibitors [46]. Many DHFR inhibitors have been synthesized by modification of the structural components of folate. For example, MTX differs by replacement of the 4-oxo with a 4-amino group and an additional N10 methyl group. There are several conserved residues within the DHFR active site [46–48] that facilitate the binding of DHFR inhibitors (Table 6).

**Table 6:** Important human DHFR residues for inhibitor binding

Functional interaction	DHFR residue
2-amino hydrogen bonding	^30^Glu
4-amino hydrogen bonding	^7^Ile
4-amino hydrogen bonding	^115^Val
N1 hydrogen bonding	^30^Glu
Pteridine ring hydrophobic interactions	^22^Leu
Pteridine ring hydrophobic interactions	^31^Phe
Pteridine ring hydrophobic interactions	^34^Phe
Benzene ring hydrophobic interactions	^22^Leu
Benzene ring hydrophobic interactions	^31^Phe
Benzene ring hydrophobic interactions	^34^Phe
Benzene ring hydrophobic interactions	^61^Pro
5-methyl hydrophobic interactions	^115^Val
9-methyl and 10-methyl hydrophobic interactions	^22^Leu

(Table formulated using information from [46–48])

An important feature for DHFR inhibition is a 2,4-diamino-substituted pyrimidine ring [49]. The 2-amino and 4-amino groups form hydrogen bonds with highly conserved residues, which orientate the pteridine ring of the inhibitor accordingly. The orientation of inhibitor binding differs from natural folate binding. Although both involve ^30^Glu, the orientation of the inhibitor allows extensive hydrogen bonding with other DHFR residues, which is not possible in the folate binding orientation. Binding of inhibitors containing 2,4-diaminopyrimidine shows the 2-amino group forms a hydrogen bond with ^30^Glu [50]. Additionally, the 4-amino group of the inhibitor forms strong hydrogen bonds with the carbonyl groups of ^7^Ile and ^115^Val [46, 50]. The protonated form of N1 forms an electrostatic interaction with ^30^Glu [47, 51].

The N8 nitrogen of the pteridine ring forms a conserved network with a water molecule, which also hydrogen bonds with ^30^Glu and ^24^Trp [46, 52]. However, comparison of pyrido [2,3-d]pyrimidines and quinazolines suggests that the presence of the N8 hydrogen bond in inhibition by pyrido [2,3-d]pyrimidines may restrict the position of any bridge substituent, such as 9 or 10 methyl groups, preventing hydrophobic interaction of these features with the DHFR active site [53, 54]. Therefore, in general quinazoline DHFR inhibitors are more potent than pyrido [2,3-d] pyrimidine analogues.

The TMACC interpretation showed variable activity for the 2-amino group, with the activity approximately evenly distributed between negative and positive activity. However, interpretation of the 4-amino group showed a greater frequency of positive activity. The interpretation showed the N1 nitrogen as generally negative for activity. This classification could possibly result from the fact that the N1 was unprotonated within most molecules of the data set. However, studies suggest the protonated form of N1 forms a salt bridge with the DHFR active site [51, 55].

Comparison of the activity of C8 atoms in quinazoline inhibitors to N8 atoms in pyrido [2,3-d]pyrimidine inhibitors assigned by the TMACC interpretation showed the C8 atom of quinazoline inhibitors to be identified as positive for activity, whilst the N8 of pyrido [2,3-d]pyrimidines was most frequently negative for activity. This interpretation reflects experimental evidence, which indicates the presence of a C8 (quinazoline) is more potent for DHFR inhibition than an N8 (pyrido [2,3-d]pyrimidine) [56].

The presence of a 5-methyl group increases inhibitor potency by forming hydrophobic interactions with ^115^Val in human DHFR [53]. Methyl groups in the 9 and 10 positions of the bridge region also improve DHFR inhibitor potency, as the environment surrounding the bridge region is generally hydrophobic. Therefore, hydrophobic interactions with ^22^Leu may be formed by these groups [47]. The benzyl ring featured in many antifolate inhibitors forms hydrophobic contacts with many residues within the DHFR active site [46, 51]. This feature is often substituted for other aromatic rings and methoxy groups to increase hydrophobic interactions.

The TMACC interpretation identified 5-methyl groups as positive for activity, consistent with experimental data, which suggests this feature is important for the inhibition of human DHFR. The interpretation also identified methyl groups in the 9 and 10 positions as positive for activity. This is consistent with the known hydrophobic interactions formed by these features within the DHFR active site [47]. The TMACC interpretation identified the methyl of methoxybenzyl groups substituted around the benzoic acid moiety of inhibitor molecules as positive for activity. This classification is supported by the hydrophobic interactions in which methoxy groups have been shown to participate within the DHFR active site [46, 51]. The TMACC interpretation of the DHFR data set identified many key structural features for DHFR inhibition. The analysis suggests that the hydrophobic groups investigated were more frequently identified as positive for activity than the hydrogen bonding groups investigated (Figure 6).

## Conclusion

Analyses of the TMACC QSARs modelled for the ACE and DHFR data sets have shown that the TMACC interpretation can identify distinctive features of a structure-activity relationship. The TMACC interpretation provided a clear and precise representation of the activity of specific groups. Amalgamation of the atomic activity values determined for such groups within a data set, showed strong correlation with experimental evidence, which shows the TMACC interpretation can produce models which accurately depict the features of a structure-activity relationship.

Overall, the TMACC interpretation modelled the ACE inhibitor structure-activity relationship highlighted important features for C-domain selective ACE inhibition. The TMACC interpretation provided a consistent representation of the structure-activity relationship present in the ACE data set. However, the insight into the structure-activity relationship of ACE inhibitors produced by the TMACC interpretation was limited by the size of the data set. To obtain a more detailed analysis of components important or detrimental to the ACE inhibitor structure-activity relationship, it would be necessary to investigate a data set which represents a comprehensive range of functional groups and structural components. Investigation of the activity of features important for C-terminal domain selective inhibition in comparison to features important for N-terminal domain selective inhibition would provide further insight into the interpretive ability of the TMACC descriptors.

An inherent weakness, due to the 2D nature of the TMACC descriptor, is insensitivity to chirality. However, the use of chirality descriptors derived from topological data may provide a solution to this limitation and may also improve the predictive ability of the QSAR models [57]. Investigation of alternative or additional atomic properties used in the TMACC descriptor would provide an insight into the properties which contribute towards activity. The effect of implementing more sophisticated partial charge calculations would be interesting, as a recent study has suggested that the method used for partial charge calculations can affect QSAR predictive accuracy [58]. Investigation of a wider range of data sets would provide further validation of the utility of the TMACC interpretation.

## Authors’ original submitted files for images

Below are the links to the authors’ original submitted files for images. Authors’ original file for figure 1 Authors’ original file for figure 2 Authors’ original file for figure 3 Authors’ original file for figure 4 Authors’ original file for figure 5 Authors’ original file for figure 6 Authors’ original file for figure 7
