# Supplementary figures and images for: Interpretable correlation descriptors for quantitative structure-activity relationships
Source: J Cheminform. 2009 Dec 24;1:22. doi: 10.1186/1758-2946-1-22 (PMC2820500; doi:10.1186/1758-2946-1-22)

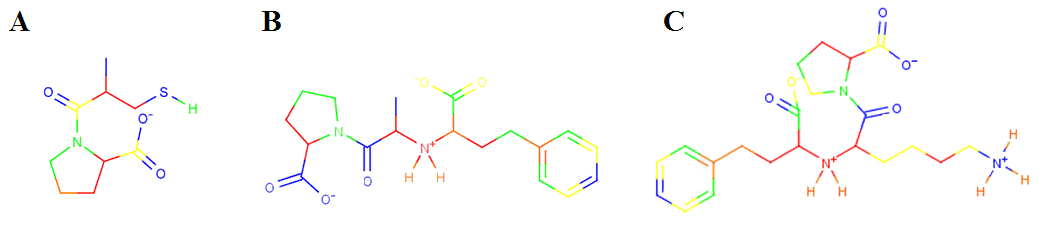

Supplement: Supplementary file 1 — Authors’ original file for figure 1 [file 13321_2009_22_MOESM1_ESM.tiff]

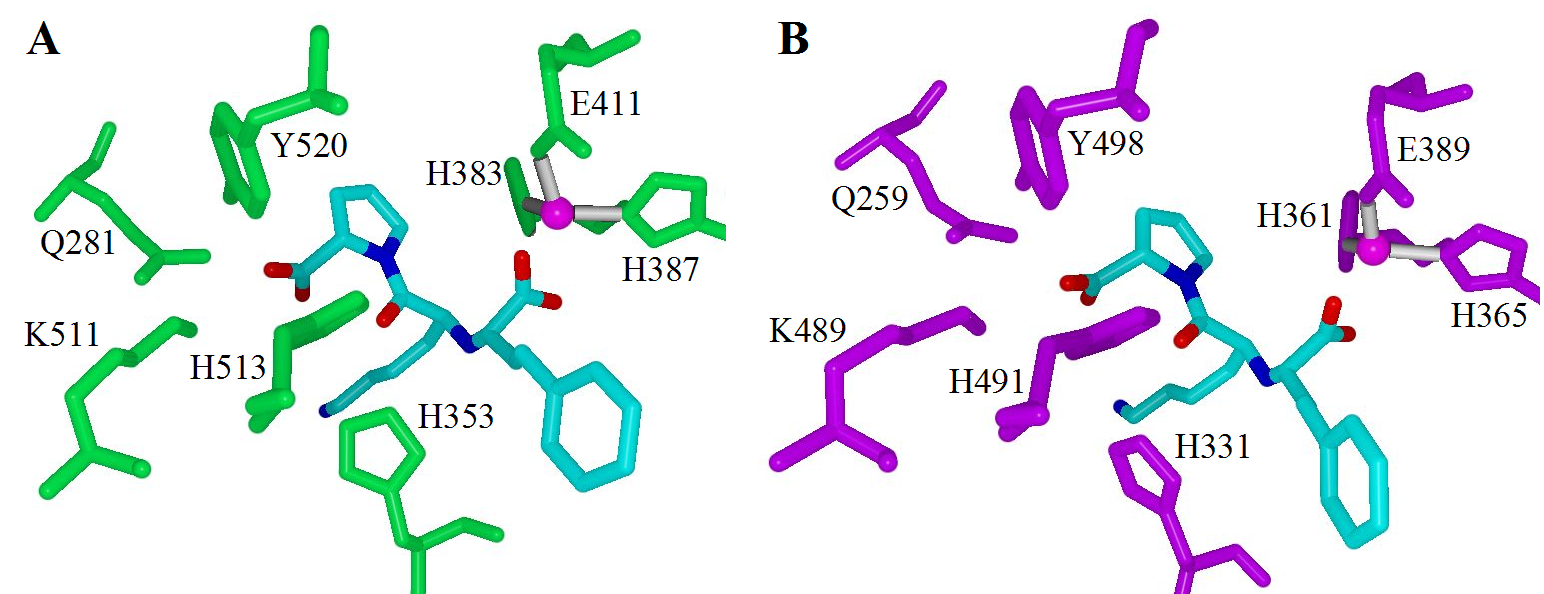

Supplement: Supplementary file 3 — Authors’ original file for figure 3 [file 13321_2009_22_MOESM3_ESM.tiff]

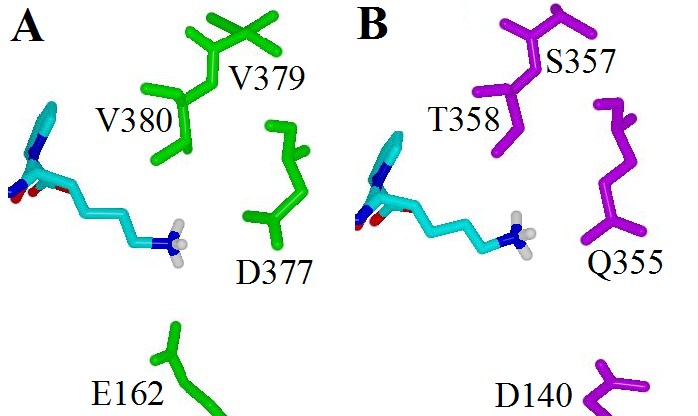

Supplement: Supplementary file 4 — Authors’ original file for figure 4 [file 13321_2009_22_MOESM4_ESM.tiff]

## Slide 1
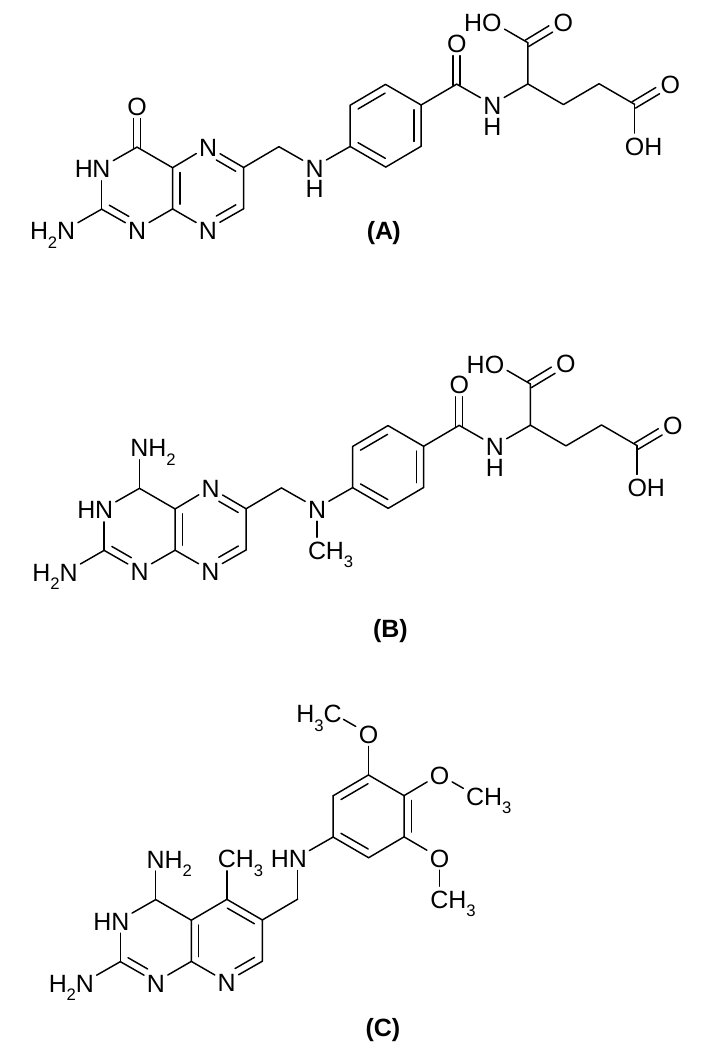

Supplement: Supplementary file 5 — Authors’ original file for figure 5 [file 13321_2009_22_MOESM5_ESM.ppt]

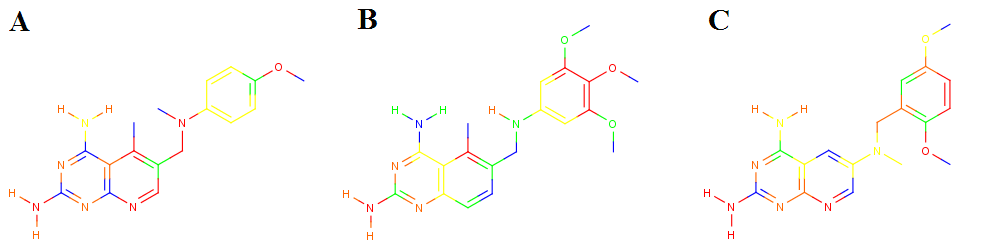

Supplement: Supplementary file 6 — Authors’ original file for figure 6 [file 13321_2009_22_MOESM6_ESM.tiff]

## Slide 1
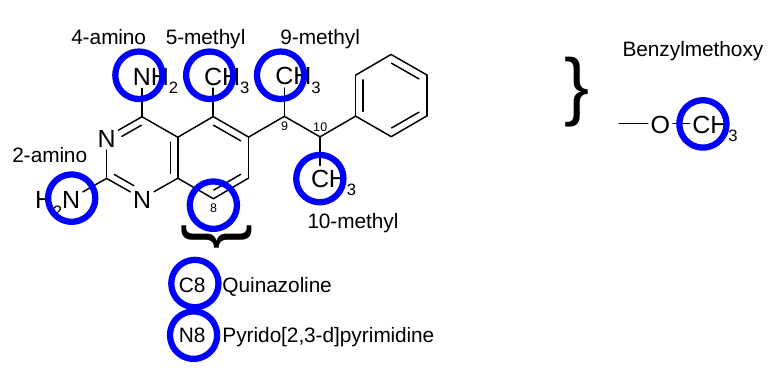

4-amino
5-methyl
9-methyl
Benzylmethoxy
}
2-amino
}
10-methyl
C8 Quinazoline
N8 Pyrido[2,3-d]pyrimidine

Supplement: Supplementary file 7 — Authors’ original file for figure 7 [file 13321_2009_22_MOESM7_ESM.ppt]
