# Supplementary material for: Interpretable correlation descriptors for quantitative structure-activity relationships
Source: J Cheminform. 2009 Dec 24;1:22. doi: 10.1186/1758-2946-1-22 (PMC2820500; doi:10.1186/1758-2946-1-22)
Supplement: Supplementary file 2 — Authors’ original file for figure 2 [file 13321_2009_22_MOESM2_ESM.ppt]

## Slide 1
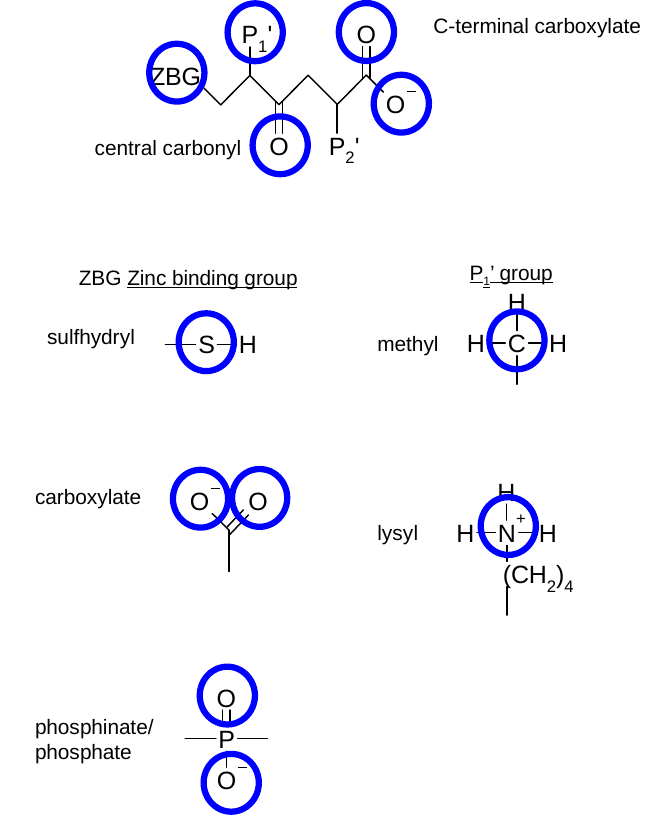

C-terminal carboxylate
central carbonyl
P1’ group
ZBG Zinc binding group
sulfhydryl
methyl
carboxylate
lysyl
phosphinate/
phosphate
